# Supplementary material for: African Swine Fever Virus pE199L Induces Mitochondrial-Dependent Apoptosis
Source: Viruses. 2021 Nov 8;13(11):2240. doi: 10.3390/v13112240 (PMC8617669; doi:10.3390/v13112240)
Supplement: Supplementary file 1 [file viruses-13-02240-s001.zip › viruses-1400662-supplementary.pdf]

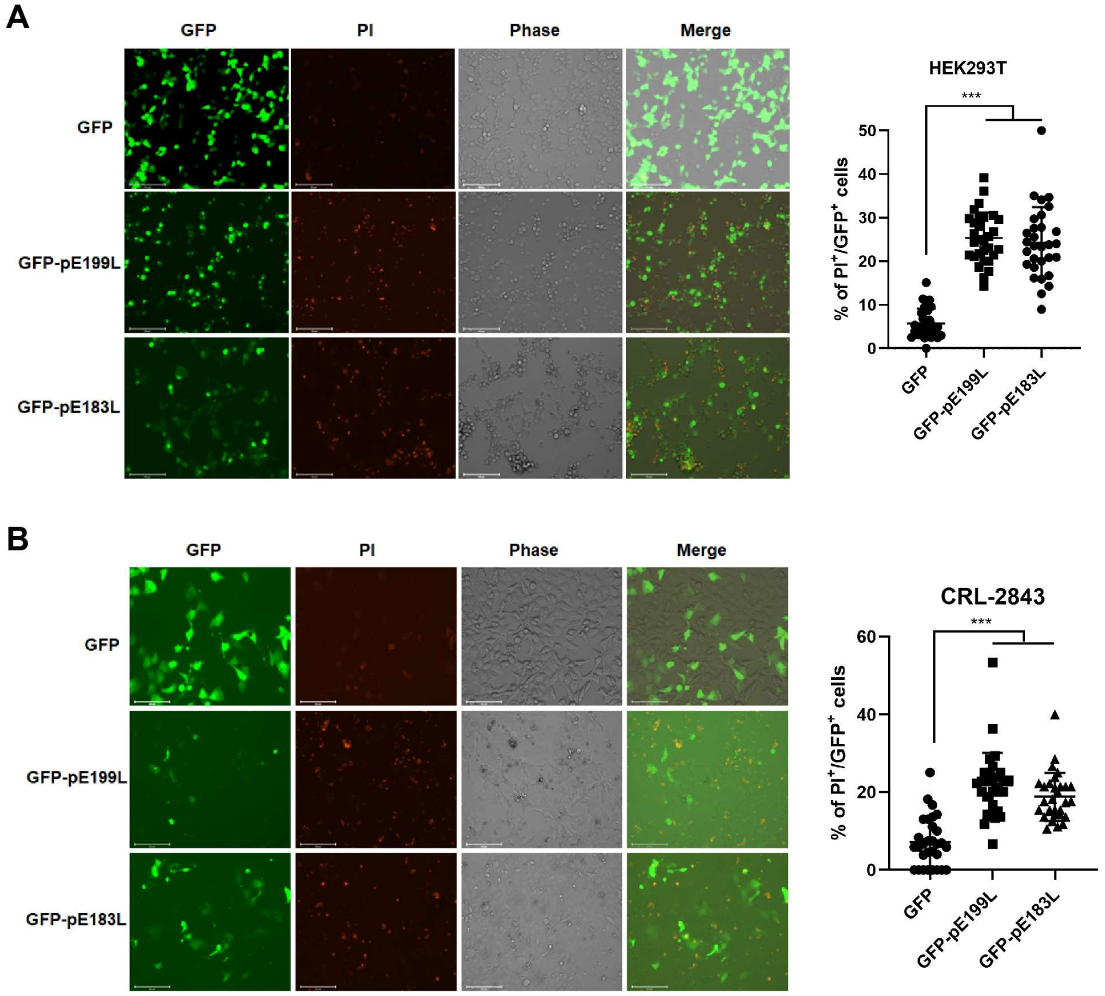

Figure S1. (**A**,**B**) Fluorescence images of PI-staining assay in GFP, GFP-pE199L or GFP-pE183L-expressing HEK293T (A) or CRL-2843 cells (B). HEK293T or CRL-2843 cells were transfected with pEGFP-C1, pGFP-E199L or pGFP-E183L plasmids for 36 h and then stained with PI for 15 min. Representative fluorescence images (left) were visualized using a fluorescence microscope (Invitrogen EVOS FL Auto 2.0) under a 20× objective. Green, GFP, GFP-pE199L or GFP-pE183L; Red, PI; Scale bar, 125 μm. The right panels showed the statistical analysis of the percentage of PI-labeling cells in GFP, GFP-pE199L or GFP-pE183L-expressing cells. PI-labeling cells from 10 fields (approximately 100 cells/ field) were counted under a 20× objective and the results were shown as mean ± SD values, n=30 from three independent experiments. The significance of the differences between the groups was determined by Brown-Forsythe and Welch ANOVA test with Dunnett T3 (A) or Mann-Whitney-test (B) (*** indicates $p < 0.001$).

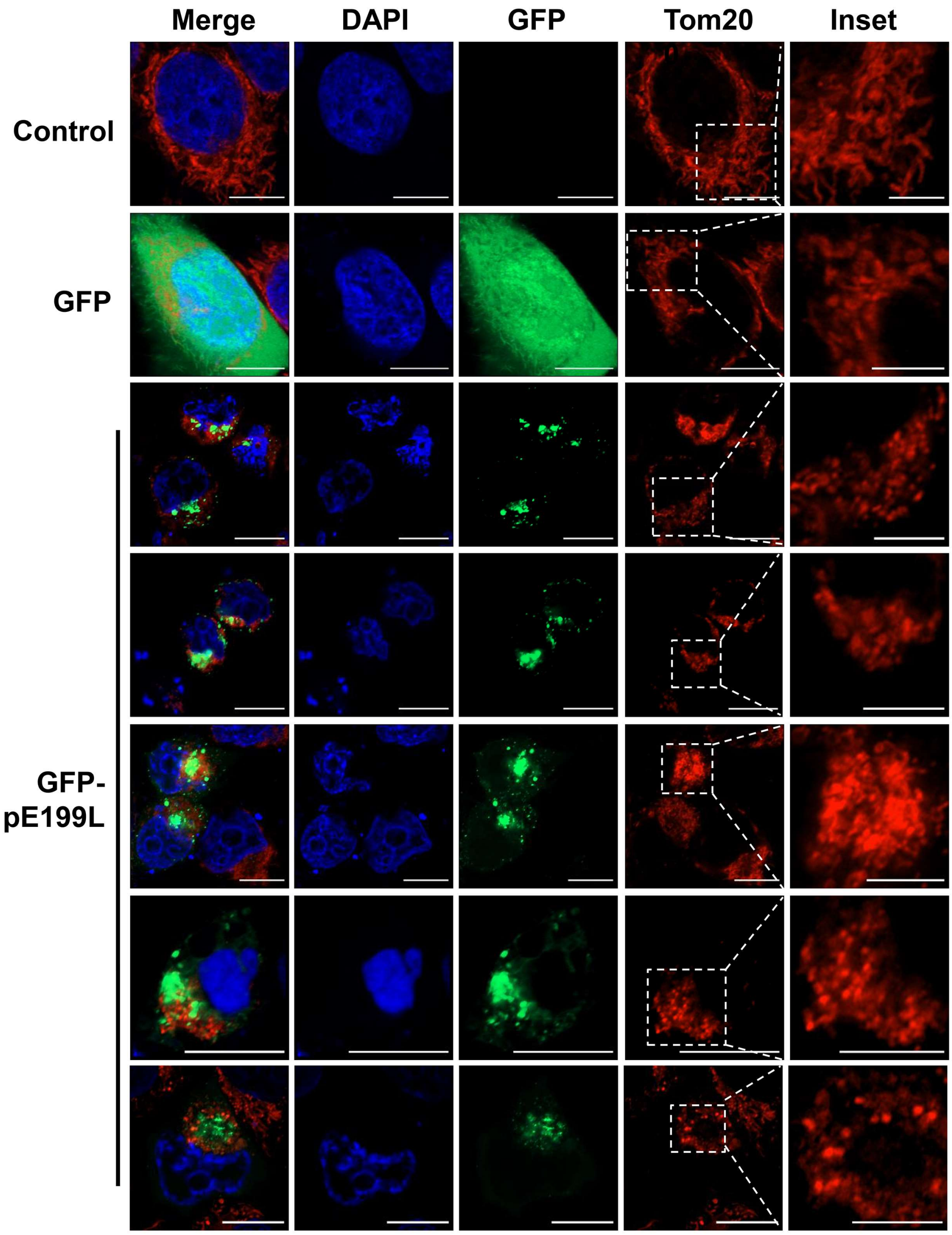

Figure S2. Representative confocal images of morphology changes of the mitochondria induced by pE199L in HeLa cells under conditions outlined in Figure 4A. Insets scale bar, 5 μm and others scale bar, 10 μm.

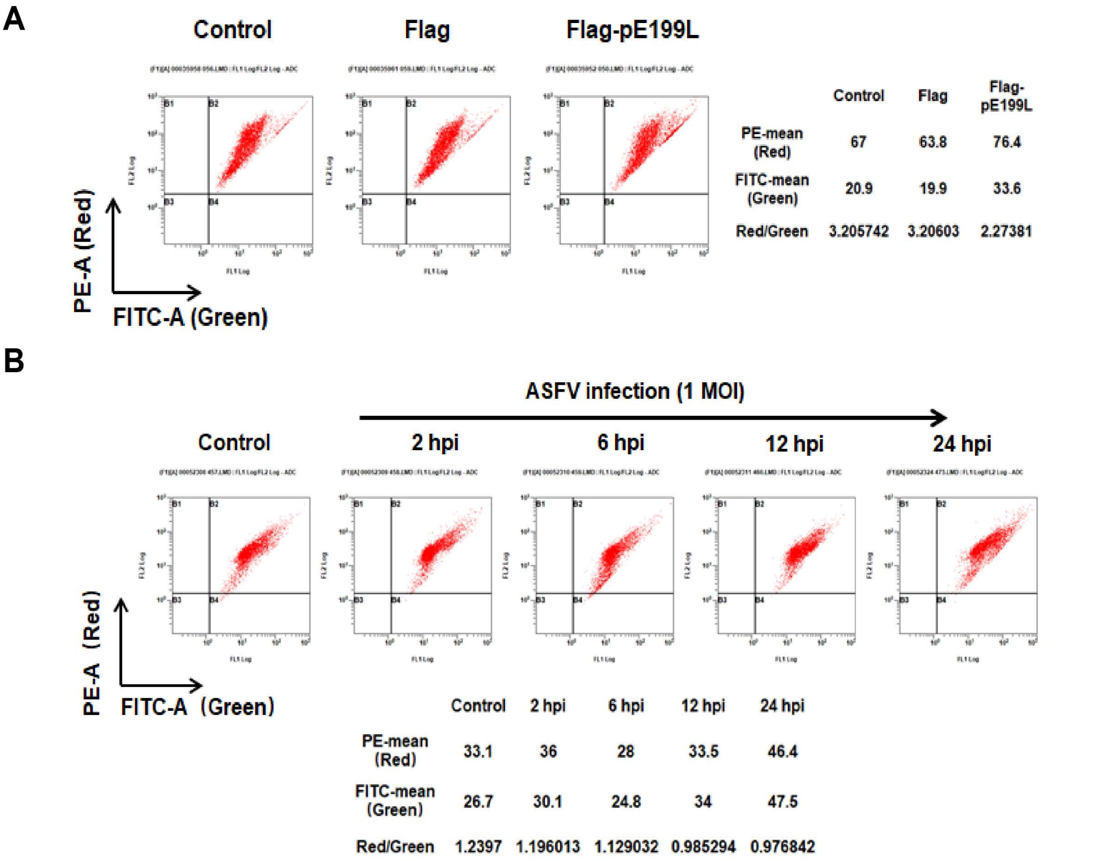

Figure S3. (**A**) Representative dot blot images of the mitochondrial membrane potential assay in HeLa cells under conditions outlined in Figure 4C. (**B**) Representative dot blot images of the mitochondrial membrane potential assay in PAMs cells under conditions outlined in Figure 6A.

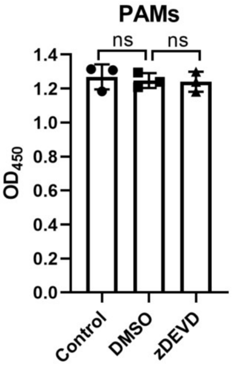

Figure S4. Detection of the cytotoxicity of z-DEVD-FMK on PAMs by WST-1 assay according to the manufacturer's instructions (Beyotime, China). PAMs were treated with z-DEVD-FMK at 200 µM or an equal volume of DMSO for 48 h, and then the cultured medium was removed and subsequently WST-1 solution (1:10 dilution) was added. Cells were incubated at 37 °C for 1 h and analyzed at 450 nm on a microplate reader (SpectraMax iD3, Molecular Devices). Data are presented as the mean ± SD, n = 3. The significance of the differences between the groups was determined by the Student's unpaired t-test with two tail (parametric test), ns indicates no statistically significant difference.
